# Decision to use e-cigarettes and associated factors among students of a university in Northern Thailand

**DOI:** 10.18332/tid/209193

**Published:** 2025-10-16

**Authors:** Civilaiz Wanaratwichit, Sunsanee Mekrungrongwong, Jutarat Rakprasit

**Affiliations:** 1 Department of Community Health, Faculty of Public Health, Naresuan University, Phitsanulok, Thailand; 2 Department of Occupational Health and Safety, Faculty of Public Health, Naresuan University, Phitsanulok, Thailand

**Keywords:** students, Thailand, decision, e-cigarettes, university

## Abstract

**INTRODUCTION:**

The use of electronic cigarettes (e-cigarettes) is spreading among adolescents, especially at higher education institutions, and it may have effects on health and learning. The objectives of this cross-sectional study were to examine the decision to use e-cigarettes and identify the associated factors among students of a university in northern Thailand.

**METHODS:**

Data were collected by using a developed questionnaire via an online system with 430 undergraduate students from a university in northern Thailand. Samples were selected by stratified random sampling. Data were analyzed using frequencies and percentages, means and standard deviations, and logistic regression at a confidence level of 0.05.

**RESULTS:**

In a sample group, the decision to use e-cigarettes was found to be 18.6%. Factors significantly associated with the decision to use e-cigarettes included receiving information about the dangers of e-cigarettes from loved ones (AOR=2.84; 95% CI: 1.20–6.71), having friends who use e-cigarettes (AOR=8.53; 95% CI: 3.41–21.37), attitudes toward e-cigarettes (AOR=3.10; 95% CI: 1.61–5.95), perceived risk of e-cigarette use (AOR=2.51; 95% CI: 1.22–5.13), and perceived benefit of avoiding e-cigarette use (AOR=2.38; 95% CI: 1.23–4.57).

**CONCLUSIONS:**

The factors associated with the decision to use e-cigarettes were found to be directly related to the students themselves, as well as their friends, acquaintances, and partners. Therefore, universities should have a policy to prevent the use of e-cigarettes by focusing on the individual level with all students. Further, activities should be organized in groups, especially among friends and partners, with a focus on changing attitudes, providing accurate information, and increasing the perceptions of e-cigarette risks as well as the benefits of avoiding e-cigarette use.

## INTRODUCTION

Smoking has adverse health effects and leads to major public health problems^1^. Currently, electronic cigarettes (e-cigarettes) have begun to replace traditional cigarettes, and are becoming increasingly popular among smokers, especially among adolescents^2^. According to the World Health Organization’s recent Global Tobacco Epidemic Report, e-cigarette manufacturers and their networks have run campaigns to mislead consumers about e-cigarettes, such as stating that e-cigarettes are less harmful and helping people to quit smoking traditional cigarettes. A report by the WHO states that such claims of e-cigarette manufacturers and their networks have no supporting evidence from neutral parties^3,4^. According to a study based on data from the Global Adult Tobacco Survey, which included 14 countries across 6 WHO regions, the highest prevalence of e-cigarette use was reported in Russia (4.39%), followed by Costa Rica (2.69%)^5^.

In the context of Thailand, recent data from the survey results of the National Statistical Office in 2021 found that roughly 78742 people in the Thai population smoked e-cigarettes, accounting for 0.14% of the population aged ≥15 years from a total of 57 million people. Among these e-cigarette smokers, 40724 were revealed to be daily smokers, 38018 were non-daily smokers, and 24050 e-cigarette smokers were aged 15–24 years. Most of them lived in Bangkok and the central region, totaling 47753 people. Meanwhile, Thai people who knew about e-cigarettes believed that they were more dangerous than traditional cigarettes (26.7%), while some thought e-cigarettes were less dangerous than traditional cigarettes (11.3%), and most thought e-cigarettes and traditional cigarettes were equally dangerous (62.0%). The data about the number and rate of Thai people who smoked e-cigarettes were a part of the Population Health Behavior Survey in 2021, which collected data from a total of 73654 households nationwide, covering a sample population of 164406 people^6^.

In particular, university students are an interesting group in terms of those who use e-cigarettes. It was found that more than half of the university students in Bangkok used e-cigarettes, with 36.6% smoking traditional cigarettes together with e-cigarettes, and 28.6% smoking only e-cigarettes^7^. Regarding the factors relating to e-cigarette use, motivation from media was found to be an important factor in deciding to use e-cigarettes^8^. According to the results of previous studies, university students still lack knowledge and viewpoints about the use of e-cigarettes. They are exposed to various media and advertisements for selling products that are easily accessible. Moreover, they believe that smoking e-cigarettes is less harmful or has less impact on health than traditional cigarettes, and they also believe that e-cigarettes help them quit or reduce the rate of traditional cigarette smoking^9-11^.

The growing interest in e-cigarettes among youth has been linked to targeted marketing strategies. E-cigarettes look like dolls, toys, and games in small sizes with brightly colored, flavored, and scented images^12^. Therefore, e-cigarettes are more interesting and spreading among teenagers. However, e-cigarettes still contain nicotine, which is a highly addictive substance. It affects the brains of teenagers, influencing their attention, learning, emotions, and impulses. It is harmful to health and life^13^, especially among university students who are in the studying process; if they use e-cigarettes, it may affect their learning and health.

The aforementioned information highlights the challenges in preventing e-cigarette use among university students. Therefore, identifying the factors influencing their decision to use e-cigarettes is critically important, particularly in university settings where existing data are limited. This research aimed to study the decision to use e-cigarettes and identify the associated factors among students at a university in northern Thailand. The findings of the study could be used as a guideline to prevent the use of e-cigarettes at universities and prevent the increase of new smokers, leading to the determination of policies that can reduce the use of e-cigarettes in the future.

## METHODS

### Study design

This research was a cross-sectional analytical study that collected data from March to December 2024.

### Population and sample

The population consisted of 24788 undergraduate students, and the samples included 436 undergraduate students at a university in northern Thailand. The sample size was determined by calculating the population proportion estimation formula with a finite population^14^ at p=0.09^15^. The sample size was 369 people, and the researchers complemented the sample size by 18%. Therefore, data were collected from a total of 436 samples.

The sample selection was conducted with the stratified selection method by classifying students into 3 strata: Health Sciences Cluster, Science and Technology Cluster, and Humanities and Social Sciences Cluster of the university. Due to limitations in accessing individual students, the originally intended random sampling method was replaced with a convenience sampling approach. Therefore, students were selected from each cluster, ensuring proportional representation of the university’s student population. The inclusion criteria were: 1) students currently studying at a Bachelor’s degree level; 2) aged ≥20 years; 3) being able to read and write Thai; and 4) students who volunteered and cooperated in the study. The exclusion criteria were those who dropped out of the university during the study period.

Data were collected via Google Forms and distributed through student network representatives from each faculty within the cluster. Consent was requested through the consent-by-action method without the need for written signatures to prevent identification. There was no time limit on answering the questionnaire. After that, the researchers checked the accuracy and completeness of the data before carrying out the analysis.

### Research instrument

This study used an online questionnaire developed from a literature review by the researchers to collect data. The developed questionnaire consisted of 6 parts as follows:

1. Personal information: This part included gender, age, faculty, education level, average monthly income, grade point average (GPA), and received information about the dangers of e-cigarette use. The questionnaire included a checklist with 7 items.
2. Social and environmental data: This part included accommodation type, exposure to e-cigarette smoke in public, having friends who use e-cigarettes, and quantity of family and friend relationships. The questionnaire included a checklist with 5 items.
3. Knowledge about e-cigarettes: The questionnaire was a yes/no checklist with 5 items. In terms of scoring, 1 point was given for a correct answer, while 0 points were given for an incorrect answer. The total score was 5 points. The criteria for dividing knowledge levels were according to Bloom^16^: <60%=low level, 60–79%=moderate level, and >80%=high level.
4. Attitudes toward e-cigarettes: This part was a Likert scale questionnaire with 5 options: strongly agree, agree, unsure, disagree, and strongly disagree. There were 8 items with positive questions being scored from 5 to 1, and negative questions being scored from 1 to 5. The attitude level was divided by the mean based on the criteria of Best^17^: mean 1.00–2.32= low level, mean 2.33–3.66=moderate level, and mean 3.67–5.00=high level.
5. Perception about e-cigarettes: This part consisted of the perceived risk of e-cigarette use, perceived severity of e-cigarette use, perceived benefit of avoiding e-cigarette use, and perceived barrier to quitting e-cigarette use, with 3 items on each sub-part for a total of 12 items. This questionnaire was a rating scale with 5 options: strongly agree, agree, unsure, disagree, and strongly disagree. Positive questions were scored from 5 to 1, and negative questions were scored from 1 to 5. The criteria for interpreting e-cigarette perception are also based on the criteria of Best^17^.
6. Decision to use e-cigarettes: This part was a checklist with 1 item. The options were the expected decision to use e-cigarettes, and the expected decision not to use e-cigarettes.

### Instrument validation

The instrument used in this study was examined for quality by finding the item-content validity index (I-CVI) from 5 experts including: social and scientific researcher, epidemiologist, research methodologist, public health experts on tobacco and drugs. The instrument passed the criterion ≥0.8^18^, and all 6 parts gained 1 point.

The instrument was tested with another group similar to the sample group, i.e. 30 undergraduate students who were studying in faculties that were not selected as a sample group. Questionnaire reliability was calculated by using the Kuder-Richardson 20 (KR-20) formula for the knowledge test, resulting in 0.72. The Cronbach’s alpha coefficient was used for testing attitudes toward e-cigarettes and perception about e-cigarettes, resulting in 0.82 and 0.71, respectively.

### Statistical analysis

Basic data were analyzed with descriptive statistics using frequencies and percentages, and means and standard deviations for analyzing personal data, knowledge, attitude, perception about e-cigarettes, and the decision to use e-cigarettes. The relationship between variables and the decision to use e-cigarettes was analyzed with binary logistic regression by enter method, and the results were interpreted with adjusted odds ratio (AOR), 95% confidence interval (CI) at a significance level of 0.05. Before conducting the logistic regression analysis, the assumptions were tested and found to meet the required criteria. Data were analyzed using IBM SPSS statistics.

### Protection of rights for the sample group

The researchers protected the sample group’s rights by requesting approval from the Human Research Ethics Committee, and the study was approved on 27 March 2024 (IRB No. P30110/2023 COE No. 089/2024). The protection of the sample group’s rights was explained through an online questionnaire.

## RESULTS

### Personal data

From the 436 sample students, 430 responded to the questionnaire, accounting for 98.6% response. According to the study results, the majority of the sample group was female (74.7%), and most of them were aged 18–20 years (63.0%). Their GPAs were mostly higher than 3.00 (59.3%), and their average monthly income was between 3001–6000 Thai Baht (53.0%). Most students received information about the dangers of e-cigarettes from social media (32.7%). They mostly lived in a dormitory or rented house (71.4%), and most had been exposed to e-cigarette smoke in a public place during the past 30 days (61.9%). The students had friends who used e-cigarettes (55.6%), and they had good relationships with their friends and family (86.0% and 95.8%, respectively).

### Knowledge, attitude, perception about e-cigarettes, and decision to use e-cigarettes

The majority of the sample group had a high level of knowledge about e-cigarettes (94.2%, mean=4.48, SD=0.66). Their attitude toward e-cigarettes was at a high level (mean=3.68, SD=0.69), perceived risk of e-cigarette use was at a high level (mean=2.55, SD=1.19), perceived severity of e-cigarette use was at a high level (mean=4.28, SD=0.75), perceived benefit of avoiding e-cigarette use was at a high level (mean=4.06, SD=0.76), and perceived barrier to quitting e-cigarette use was at a moderate level (mean=3.34, SD=0.76). Most sample students decided not to use e-cigarettes (81.4%), while few students decided to use e-cigarettes (18.6%) (Table 1).

**Table 1:** Participants’ knowledge, attitude, perception about e-cigarettes, and decision to use e-cigarettes (N=430)

*Variables*	*n*	*%*
**Knowledge about e-cigarettes**		
Low level	6	1.4
Moderate level	19	4.4
High level	405	94.2
Mean=4.48, SD=0.66		
**Attitude toward e-cigarettes**		
Low level	11	2.5
Moderate level	165	38.4
High level	254	59.1
Mean=3.68, SD=0.69		
**Perceived risk of e-cigarette use**		
Low level	74	17.2
Moderate level	143	33.3
High level	213	49.5
Mean=2.55, SD=1.19		
**Perceived severity of e-cigarette use**		
Low level	9	2.1
Moderate level	45	10.5
High level	376	87.4
Mean=4.28, SD=0.75		
**Perceived benefit of avoiding e-cigarette use**		
Moderate level	108	25.1
High level	322	74.9
Mean=4.06, SD=0.76		
**Perceived barrier to quitting e-cigarette use**		
Moderate level	256	59.5
High level	174	40.5
Mean=3.34, SD=0.76		
**Decided to use e-cigarettes**		
Decided not to use e-cigarettes	350	81.4
Decided to use e-cigarettes	80	18.6

### Factors associated with decision to use e-cigarettes

After testing the assumptions, variables were included in the binary logistic regression analysis. Subsequently, variables with a significance level of ≤0.20 were selected for inclusion in the multivariate logistic regression model. Seven variables were retained: gender, academic performance, information received from partners, having friends who use e-cigarettes, attitudes, perceived risk of e-cigarette use, and perceived benefit of avoiding e-cigarette use. The study results showed 5 factors significantly associated with the decision to use e-cigarettes at the 0.05 level: 1) receiving information about the dangers of e-cigarettes from partners; 2) having friends who use e-cigarettes; 3) attitude toward e-cigarettes; 4) perceived risk of e-cigarette use; and 5) perceived benefit of avoiding e-cigarette use. For each factor, the students who did not receive information about the danger of e-cigarettes from intimate partners had a 2.84-fold higher chance of deciding to use e-cigarettes than the students who receive information about the danger of e-cigarettes from intimate partners (AOR=2.84; 95% CI: 1.20–6.71). The students with friends who used e-cigarettes had an 8.53-fold higher chance of deciding to use e-cigarettes than the students not having friends who used e-cigarettes (AOR=8.53; 95% CI: 3.41–21.37). The students with a low level of correct attitude toward e-cigarettes were 3.10 times more likely to decide to use e-cigarettes than the students with a high level of correct attitude toward e-cigarettes (AOR=3.10; 95% CI: 1.61–5.95). The students with a low level of perceived risk of e-cigarette use were 2.51 times more likely to decide to use e-cigarettes than the students with a high level of perceived risk of e-cigarette use (AOR=2.51; 95% CI: 1.22–5.13). Finally, the students with a moderate level of perceived benefit of avoiding e-cigarette use were 2.38 times more likely to decide to use e-cigarettes than the students with a high level of perceived benefit of avoiding e-cigarette use (AOR=2.38; 95% CI: 1.23–4.57). These 5 independent variables could co-predict the variation of the dependent variables about university students’ decisions to use e-cigarettes by 41.9% (Nagelkerke R^2^=0.419) (Table 2).

**Table 2:** Factors associated with decision to use e-cigarettes among students of a university in Northern Thailand (N=430)

*Variables*	*OR (95% CI)*	*AOR (95% CI)*	*p*
**Sex**			
Male	2.21 (1.32–3.69)	1.03 (0.54–1.95)	0.927
Female ®	1	1	
**Cumulative grade point average**			
0–2.50	2.27 (1.06–4.88)	1.40 (0.58–3.38)	0.453
2.51–3.00	3.40 (1.59–7.77)	1.86 (0.76–4.55)	0.175
3.01–3.50	4.27 (1.90–9.57)	1.74 (0.66–4.61)	0.266
3.51–4.00 ®	1	1	
**Receiving information about the dangers of e-cigarettes from partners**			
Not receiving	2.76 (1.39–5.48)	2.84 (1.20–6.71)	0.017
receiving ®	1	1	
**Having friends who use e-cigarettes**			
Having	13.83 (5.86–32.61)	8.53 (3.41–21.37)	<0.001
Not having ®	1	1	
**Attitude toward e-cigarettes**			
Low level	5.54 (3.16–9.71)	3.10 (1.61–5.95)	0.001
Moderate level	20.48 (5.52–75.93)	3.40 (0.78–14.73)	0.103
High level ®	1	1	
**Perceived risk of e-cigarette use**			
Low level	3.48 (1.70–7.14)	2.51 (1.22–5.13)	0.012
Moderate level	4.82 (2.64–8.77)	2.01 (0.87–4.66)	0.102
High level ®	1	1	
**Perceived benefit of avoiding e-cigarette use**			
Moderate level	4.44 (2.66–7.42)	2.38 (1.23–4.57)	0.010
High level ®	1	1	

AOR: adjusted odds ratio. ® Reference categories.

## DISCUSSION

Most sample students decided not to use e-cigarettes (81.4%). It could be debated that most university students decided not to use e-cigarettes probably because e-cigarettes are illegal. At present, Thailand has begun to strictly enforce the law to arrest those who possess e-cigarettes and smoke them. It can be observed from the results of previous studies that students are afraid of the law, specifying that those who possess e-cigarettes are guilty^19^. Therefore, law enforcement is important. If there are comprehensive regulations, it will likely result in a decrease in e-cigarette use^20^. However, the number of students with the expectation to use e-cigarettes is almost 20% (n=80), which is still considered a large number. These findings underscore the necessity for universities to establish and enforce explicit policies aimed at preventing e-cigarette use. In addition, student groups should be strongly encouraged to critically reconsider their choices in order to avoid initiating e-cigarette use. The results of this study are consistent with a previous study that found most students have a low level of intention to use e-cigarettes^21^.

The factors associated with the decision to use e-cigarettes were receiving information about the dangers of e-cigarettes from intimate partners, having friends who use e-cigarettes, attitudes toward e-cigarettes, perceived risk of e-cigarette use, and perceived benefit of avoiding e-cigarette use. It can be observed that these factors are both internal and external, in line with the concept of decision-making that the factors influencing people’s decisions are internal and external to the individual^22^.

Interestingly, the external factors that make students decide to use e-cigarettes are from people close to them, namely friends and intimate partners. The results of the study showed that the students with friends who used e-cigarettes were 8.53 times more likely to decide to use e-cigarettes than the students who did not have friends who used e-cigarettes. It can be considered that, in university life, students spend most of their time with friends, both studying in the classroom and participating in university activities together. Friends may tend to have similar interests. Being close to friends and seeing their friends’ behavior of using e-cigarettes increases the chance of deciding to use e-cigarettes like their friends^23^. Using e-cigarettes like their friends is a way to maintain relationships with the group of friends, and it is a way for students to socialize^24^. Thus, it can be said that using e-cigarettes is for social benefit^25^. This finding is consistent with previous studies found that youths have friends who smoke is a factor that positively affects e-cigarette use^9,26,27^. In addition, the study found that having close friends who used e-cigarettes was associated with youths’ e-cigarette use^28^. This finding is consistent with a study among university students in Palestine, which revealed that cigarette use was significantly associated with having peers and mothers who smoke^29^.

Additionally, the study found that students who did not receive information about the dangers of e-cigarettes from their intimate partners were 2.84 times more likely to decide to use e-cigarettes compared to those who did receive such information. This finding may be explained to the nature of intimate relationships, which typically involve frequent communication, mutual trust, and emotional reliance. Information exchanged within these close relationships tends to exert a substantial influence, especially when it concerns potential health risks such as the harms of e-cigarettes. This finding aligns with the Health Belief Model (HBM) that explains interpersonal influence in inducing or performing behaviours^30^. Therefore, when students receive clear warnings from their intimate partners about the dangers of e-cigarettes, they may be more motivated to avoid using them. Interpersonal influence, particularly from intimate partners, thus plays a crucial role in shaping health-related decision-making and behavior regarding e-cigarette use.

The important findings of the study revealed that students’ intimate partners and people close to them had a significant influence on their decision to use e-cigarettes, both by showing them how to use e-cigarettes and by providing information about e-cigarettes. Therefore, university-level strategies to prevent e-cigarette use should emphasize the organization of inclusive group activities that engage students, particularly through interactions with close friends and intimate partners.

Internal factors affect university students’ decision to use e-cigarettes. The results of the study showed that the sample group with a low level of correct attitude toward e-cigarettes were 3.10 times more likely to decide to use e-cigarettes than those with a high level of correct attitude toward e-cigarettes. It can be discussed that students with incorrect attitudes toward e-cigarettes feel that using e-cigarettes is safe and not harmful to health. In addition, e-cigarettes have modern and attractive appearances, which may impact the decision to use e-cigarettes. Therefore, having incorrect beliefs will affect the decision to use e-cigarettes^31^. Many students tend to believe that using e-cigarettes does not hurt their health^32^. This study finding is consistent with previous studies on youth in Uttaradit Province, which found that attitude toward smoking e-cigarettes was a factor related to e-cigarette smoking behavior among youths^27,28,33,34^. It was found that university students with positive attitudes toward e-cigarettes were more likely to use e-cigarettes. This finding was not different from a study in China, which found that the main factor motivating university students to use e-cigarettes was the belief that e-cigarettes were less harmful or harmless^35^. In addition, this finding is consistent with previous studies in Australia, which found that young Australians have a good attitude toward e-cigarettes by using e-cigarettes for managing their psychological distress^36^. The results of this study reflect that attitude towards e-cigarettes is very important, and the attitudes of university students should be modified properly to prevent e-cigarette use.

In addition, it was found that students with a low level of perceived risk of e-cigarette use were 2.51 times more likely to decide to use e-cigarettes than those with a high level of perceived risk of e-cigarette use. It can be discussed that students with a low level of perceived risk of e-cigarette use may not understand various situations that lead to the risk of using e-cigarettes, making them unable to avoid those situations. Therefore, a low level of perceived risk of e-cigarette use is a higher risk factor for deciding to use e-cigarettes than a high level of perceived risk of e-cigarette use. This finding is consistent with a previous study that found students with a low level of perceived risk were more likely to use e-cigarettes^27^. Moreover, the results of this study also showed that the sample group with a moderate level of perceived benefits of avoiding e-cigarette use was 2.38 times more likely to decide to use e-cigarettes than those with a high level of perceived benefits of avoiding e-cigarette use. Thus, it can be reasoned that students with low perceived benefits of avoiding e-cigarette use have a higher chance of deciding to use e-cigarettes. The more they perceive the benefits of avoiding e-cigarette use, the more they understand and are able to decide to avoid using e-cigarettes. This is in line with the concept of perceived benefits of disease prevention according to the Health Belief Model^30^, which states that, if individuals perceive the benefits of practice by accepting it as a good practice, this perception will motivate them to decide to practice. The study finding highlights the need to create a better perception of both the risks of e-cigarette use and the benefits of avoiding e-cigarette use among students as a strategy to prevent the decision to use e-cigarettes.

### Strengths and limitations

A key strength of this study lies in the diversity of the sample, which included students from all academic disciplines rather than focusing on a single faculty or department. This study was conducted by a cross-sectional design, which restricts the ability to establish causal relationships between variables and the decision to use e-cigarettes. Initially, we planned to select the participants by random sampling. However, the participant recruitment was replaced by convenience sampling due to difficulties in accessing student data. Selection bias might have occurred, as the questionnaire addressed sensitive issues related to substance use and legal implications, which may have affected the willingness of participants to complete the questionnaire.

## CONCLUSIONS

The findings of this study indicated a substantial likelihood that university students may make a decision to use e-cigarettes in the future. This underscores the need for universities to establish clear and actionable policies aimed at preventing e-cigarette use among students. The decision to use e-cigarettes was influenced by both individual attitudes and perceptions, as well as close interpersonal relationships particularly with friends and intimate partners.

To address this issue, universities should implement proactive and evidence-based strategies targeting individual-level interventions across student populations. These efforts should focus on reshaping attitudes toward e-cigarettes, enhancing awareness of health risks, and emphasizing the benefits of abstaining from use. Such strategies can help prevent the new users among students at risk.

## Data Availability

The data supporting this research are available from the authors on reasonable request.
